# A Nonsurgical Treatise on Diseases of the Prostate Gland and Adnexa

**Published:** 1904-02

**Authors:** 


A Nonsurgical Treatise on Diseases of the Prostate Gland and Adnexa. By George Whitfield Overall, M. D., formerly Professor of Physiology in the Memphis Hospital Medical College. Duodecimo, pages 217. Illustrated. Chicago: Marsh & Grant Co. 1903.

There is some good in this book which cannot be spoiled by the author's faddism or his mistaken idea regarding stricture of the prostatic urethra. One of the good features is that he approaches the subject in a methodic manner; another is that the material is based largely on his own experience, and is illustrated by case reports. He opens with a brief description of the anatomy and function of the prostate. The diseases of the organ are taken up in order, their symptoms concisely stated, causation, probable duration and prognosis clearly and lucidly outlined. Treatment is also given, but this portion is useless except to one who is immersed in the belief that in electrolysis and cataphoresis lie the cure of all prostatic disease. Dr. Overall freely admits there are some cases where operation is justifiable, but they are so few and far between that they apparently do not merit mention.

From the standpoint of the chronic electrotherapist the work is complete; and although one might question Dr. Overall's persistent antagonism to operative procedures for hypertrophied prostate in favor of his equally persistent advocacy of electrolysis for pretty nearly every ill of the organ, yet his work shows him to be thoroughly in earnest,—even if slightly misguided and overburdened with enthusiasm for his methods. It is unfortunate that Dr. Overall, who is really a clever man and an earnest student, should be among the last to emerge from the electrolytic trance. His statement that organic stricture is never found in the prostatic urethra is a mis-statement of fact, unintentional, of course. Dr. Overall has been merely unfortunate in not seeing stricture thus placed, for such strictures do exist. Fuller, of New York, has reported them; so has Park, of Buffalo.

